# Quantifying Suicide Risk in Prostate Cancer: A SEER-Based Predictive Model

**DOI:** 10.1007/s44197-025-00384-z

**Published:** 2025-03-20

**Authors:** Jiaxing Du, Fen Zhang, Weinan Zheng, Xue Lu, Huiyi Yu, Jian Zeng, Sujun Chen

**Affiliations:** 1 Department of Pediatric Intensive Care Unit, Dongguan Children’s Hospital Affiliated to Guangdong Medical University, Dongguan, China; 2 Dongguan Children’s Hospital Affiliated to Guangdong Medical University, Dongguan, China

**Keywords:** Prostate cancer, Suicide, Nomogram, SEER, Risk prediction

## Abstract

**Background:**

Prostate cancer patients have a significantly higher risk of suicide compared to the general population. This study aimed to develop a nomogram for identifying high-risk patients and providing empirical evidence to guide effective intervention strategies.

**Methods:**

We analyzed data from 176,730 prostate cancer patients diagnosed between 2004 and 2021, sourced from the Surveillance, Epidemiology, and End Results (SEER) database. Patients were randomly allocated to training (*n* = 123,711) and validation (*n* = 53,019) cohorts in a 7:3 ratio. Feature selection was conducted using the Least Absolute Shrinkage and Selection Operator (LASSO), followed by model construction with Cox proportional hazards regression. The results were visualized using nomogram. Model performance was evaluated with time-dependent receiver operating characteristic (ROC) curves, concordance index (C-index), and internal validation.

**Results:**

Multivariate analysis identified seven independent predictors of suicide. The nomogram demonstrated favorable discriminative capability in both cohorts, with C-index of 0.746 and 0.703 for the training and bootstrapped validation cohorts. Time-dependent ROC analysis indicated strong accuracy in predicting suicide risk. Calibration plots displayed high concordance between predicted probabilities and actual outcomes, Kaplan-Meier analysis confirmed the model’s significant discriminative ability among risk groups.

**Limitations:**

This retrospective study, based on SEER data, lacks detailed clinical and mental health information. Additionally, potential coding errors and reporting biases may affect the accuracy of the results.

**Conclusion:**

We developed a applicable nomogram for the individualized quantification of suicide risk in prostate cancer patients. This model provides clinicians with a robust tool for identifying high-risk patients and implementing timely interventions.

**Supplementary Information:**

The online version contains supplementary material available at 10.1007/s44197-025-00384-z.

## Introduction

Prostate cancer is one of the most prevalent malignant neoplasms in the male population worldwide. According to the 2020 global cancer statistics, the incidence of prostate cancer approached 1.4 million new cases, with mortality figures nearing 380,000 [1]. Despite continuous advancements in diagnostic and therapeutic modalities, the quality of life and psychological well-being of patients remain critical areas of scientific inquiry. A substantial body of literature indicates that oncology patients have a significantly higher risk of suicide compared to the general population, with those diagnosed with prostate cancer showing particular vulnerability [2–4]. These individuals face a complex array of challenges, including disease-related physical symptoms, iatrogenic effects of treatment, and psychosocial stressors. Such factors may precipitate negative affective states, including depression and anxiety, thereby potentially exacerbating suicidal ideation and behavior [5, 6].

A comprehensive meta-analysis of observational studies revealed that men diagnosed with prostate cancer have an overall relative risk of suicide increased to 2.01 (95% CI: 1.52–2.64) compared to those without prostate cancer during the first year after diagnosis [7]. It is noteworthy that the suicide risk among this patient population exhibits significant geographic variability. For instance, epidemiological studies in the United States have demonstrated a 15% increase in suicide risk for prostate cancer patients relative to the general population [8], while Swedish cohort studies report a relatively larger increase to 2.6 times [9]. These disparities may be attributed to a complex interplay of factors, including cultural milieu, healthcare system infrastructure, and the robustness of social support networks.

The etiology of suicide risk in prostate cancer patients is multifactorial, involving physiological and psychological factors [10]. These etiological factors include demographic variables (e.g., age and ethnicity), socioeconomic indicators (e.g., educational attainment and income level), clinical parameters (e.g., disease stage and therapeutic approach), and psychiatric comorbidities [11, 12]. However, the current body of research on suicide risk in this patient population has several methodological limitations. Primarily, the majority of studies focus on identifying potential risk factors, lacking robust predictive models for quantifying individual suicide risk. Additionally, extant research often struggles to explain the temporal dynamics of suicide risk, particularly during various post-diagnostic intervals.

In light of these research gaps, the development of accurate and reliable predictive models is imperative for enhanced identification and prevention of suicidal behavior in prostate cancer patients. In recent years, machine learning methodologies have emerged, showing potential superiority over traditional statistical approaches in medical prognostication [13]. Among these, nomograms have garnered particular attention as intuitive and individualized risk assessment tools, showing promising applications across various oncological domains [14]. Based on these considerations, our study utilizes the Surveillance, Epidemiology, and End Results (SEER) database to develop, for the first time, a comprehensive suicide risk prediction model for prostate cancer patients incorporating multidimensional factors. We employ advanced machine learning algorithms to optimize the model’s predictive accuracy and account for the temporal dynamics of suicide risk. Our analysis identified seven independent predictors of suicide, encompassing demographic characteristics, socioeconomic indicators, interpersonal factors, and tumor-related variables. We propose that this predictive model will provide clinicians with a practical tool for identifying high-risk patients and implementing timely interventions, potentially improving the quality of life and long-term prognosis of individuals with prostate cancer.

## Materials and Methods

### Data Source and Design

This study employed a population-based retrospective cohort design using data from SEER database. The SEER database serves as a comprehensive epidemiological resource, enabling detailed analyses of cancer incidence, treatment modalities, and long-term survival outcomes. Data were extracted using SEER*Stat Version 8.4.3, which encompasses records from 17 registries representing approximately 34.6% of the U.S. population [15], ensuring robust external validity and generalizability. The dataset spans includes both publicly accessible variables (e.g., sociodemographic characteristics, tumor-specific parameters, longitudinal follow-up data) and restricted-access variables (e.g., radiotherapy and chemotherapy protocols).

This study uses de-identified data from the publicly accessible SEER database, ensuring no personally identifiable information and protecting patient privacy. According to the U.S. Department of Health and Human Services (HHS) guidelines (https://ori.hhs.gov/content/chapter-3-The-Protection-of-Human-Subjects-45-crf-46102-protection-human-subjects), the use of de-identified data does not involve human participants, as there is no collection of identifiable private information or direct interaction with participants. Additionally, JXD, a member of this study, has received formal authorization to access and use SEER data in compliance with SEER’s data access policies. As a result, the study qualifies for IRB exemption and does not require institutional review board approval, in accordance with SEER/National Cancer Institute (NCI) guidelines.

### Study Population

**Fig. 1:**
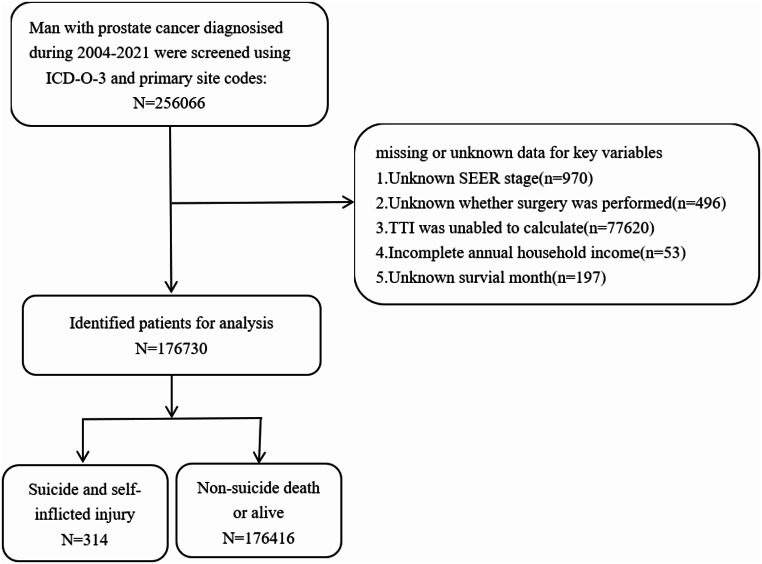
Flow chart. Illustration of patient inclusion. ICD-O-3, International Classification of Disease for Oncology (third edition); SEER, Surveillance, Epidemiology, and End Results; TTI, time to treatment initiation

The initial cohort comprised 256,066 male patients diagnosed with prostate cancer between January 1, 2004, and December 31, 2021, as identified in the SEER database. To ensure robust analyses, patients with missing or unknown data for variables and those with unknown survival times were excluded. The process of patient selection is delineated in the flow diagram presented as Fig. 1. The final analytic sample included 176,730 male patients with prostate cancer. This refined cohort was used to investigate the primary outcome: mortality due to suicide and self-inflicted injury following prostate cancer diagnosis. Variables were categorized to facilitate comprehensive analyses and to account for potential confounding factors. Age was stratified into ≤ 64 years and > 64 years. Marital status was classified into three categories: married, separated/divorced/widowed (SDW), and single. Cancer stage was categorized as localized, regional, or distant. Geographical factors were addressed by classifying patients’ residences as either metropolitan or non-metropolitan counties. Socioeconomic status was approximated using annual household income, stratified into three tiers: <$74,999, $75,000–$89,999, and >$90,000.

### Statistical Analysis

Statistical analyses were conducted using R software, version 4.4.1 (https://cran.r-project.org/bin/windows/base/old/4.4.1/). The dataset of 176,730 prostate cancer patients was divided into a training set (*n* = 123,711) and a validation set (*n* = 53,019) in a 7:3 ratio using the “create Data Partition” function from the “caret” package in R (https://cran.r-project.org/web/packages/caret/index.html). Continuous variables were analyzed with Student’s t-tests, and categorical variables were compared using chi-square tests or Fisher’s exact tests to ensure rigorous statistical evaluation. Feature selection and model construction were performed using the training set, while the validation set was utilized to evaluate the model’s performance.

### Feature Selection and Model Construction

Feature selection was conducted using the Least Absolute Shrinkage and Selection Operator (LASSO), a regularized regression technique that penalizes model complexity, generating sparse solutions by retaining only the most significant predictors. LASSO was chosen for its efficiency in handling multicollinearity and high-dimensional data, ensuring the robustness of the selected features [16]. Following feature selection, Cox proportional hazards regression was employed to construct the predictive model. This method relates multiple risk variables to survival time, facilitating the identification of independent predictors of suicide among prostate cancer patients. Nomograms were constructed based on seven optimal predictors identified during model construction, providing personalized predictions for identifying and stratifying patients at high risk of suicide.

### Model Evaluation

To evaluate the model’s accuracy in predicting long-term suicide risk, time-dependent receiver operating characteristic (ROC) curves were employed, and the area under the ROC curve (AUC) was calculated to demonstrate predictive accuracy. Calibration plots were generated to assess the agreement between predicted probabilities and actual outcomes, demonstrating high concordance and reliability of the model predictions. Kaplan-Meier survival analysis was performed to compare suicide survival curves between high- and low-risk groups, as defined by the nomogram-derived risk scores. These comprehensive methods ensured the robustness and clinical applicability of the predictive model in a clinical setting.

## Results

### Baseline Characteristics

A total of 176,730 prostate cancer patients were included in this study, with 123,711 (70.0%) in the training cohort and 53,019 (30.0%) in the validation cohort. The study population consisted of 22,589 (12.8%) Black, 140,047 (79.3%) White, and 14,094 (7.9%) patients of other races. No statistically significant differences were observed between the training and validation cohorts regarding baseline characteristics, as shown in Table 1. Among all participants, 314 (0.18%) died by suicide.

**Table 1:** Baseline clinicopathological characteristics of patients in training and Validation cohorts (*N* = 176730)

Variables	Total	Training Cohort *N* = 123,711		Validation Cohort *N* = 53,019		*P*-value
		*N*	%	*N*	%	
**Age(years)**, **n(%)**						0.537
≤ 64	75,019	52,454	42.4%	22,565	42.6%	
> 64	101,711	71,257	57.6%	30,454	57.4%	
**Race**, **n(%)**						0.198
Black	22,589	15,817	12.8%	6772	12.8%	
White	140,047	98,122	79.3%	41,925	79.1%	
Others	14,094	9772	7.9%	4322	8.2%	
**Stage**, **n(%)**						0.352
Localized	129,952	91,069	73.6%	38,883	73.3%	
Regional	32,061	22,409	18.1%	9652	18.2%	
Distant	14,717	10,233	8.3%	4484	8.5%	
**Surgery**, **n(%)**						0.074
Not recommend	81,782	57,250	46.3%	24,532	46.3%	
Recommend but not performed	5395	3851	3.1%	1544	2.9%	
Performed	89,553	62,610	50.6%	26,943	50.8%	
**Annual household income**, **n(%)**						0.400
<$74,999	52,143	36,606	29.6%	15,537	29.3%	
$80,000-$90,000	56,489	39,544	32.0%	16,945	32.0%	
>$90,000	68,098	47,561	38.4%	20,537	38.7%	
**Marital status**, **n(%)**						0.231
SDW	22,352	15,572	12.6%	6780	12.8%	
Maried	134,190	94,074	76.0%	40,116	75.7%	
Single	20,188	14,065	11.4%	6123	11.5%	
**Residence**, **n(%)**						0.748
Non-metropolitan	24,061	16,821	13.6%	7240	13.7%	
Metropolitan	152,669	106,890	86.4%	45,779	86.3%	
**FMN**, **n(%)**						0.931
No	16,321	11,430	9.2%	4891	9.2%	
Yes	160,409	112,281	90.8%	48,128	90.8%	
**Radiaotherapy**, **n(%)**						0.755
No/Unknown	98,632	69,012	55.8%	29,620	55.9%	
Yes	78,098	54,699	44.2%	23,399	44.1%	
**Chemotherapy**, **n(%)**						0.126
No/Unknown	173,784	121,687	98.4%	52,097	98.3%	
Yes	2946	2024	1.6%	922	1.7%	
**TTI(days)**	75(32,98)	75(32,98)		75(31,98)		0.196

Abbreviations SDW, Separated/Divorced/Widowed; FMN, First Malignant Neoplasm; TTI, Time of Treatment Initiation

### Variables and Nomogram Development

**Fig. 2:**
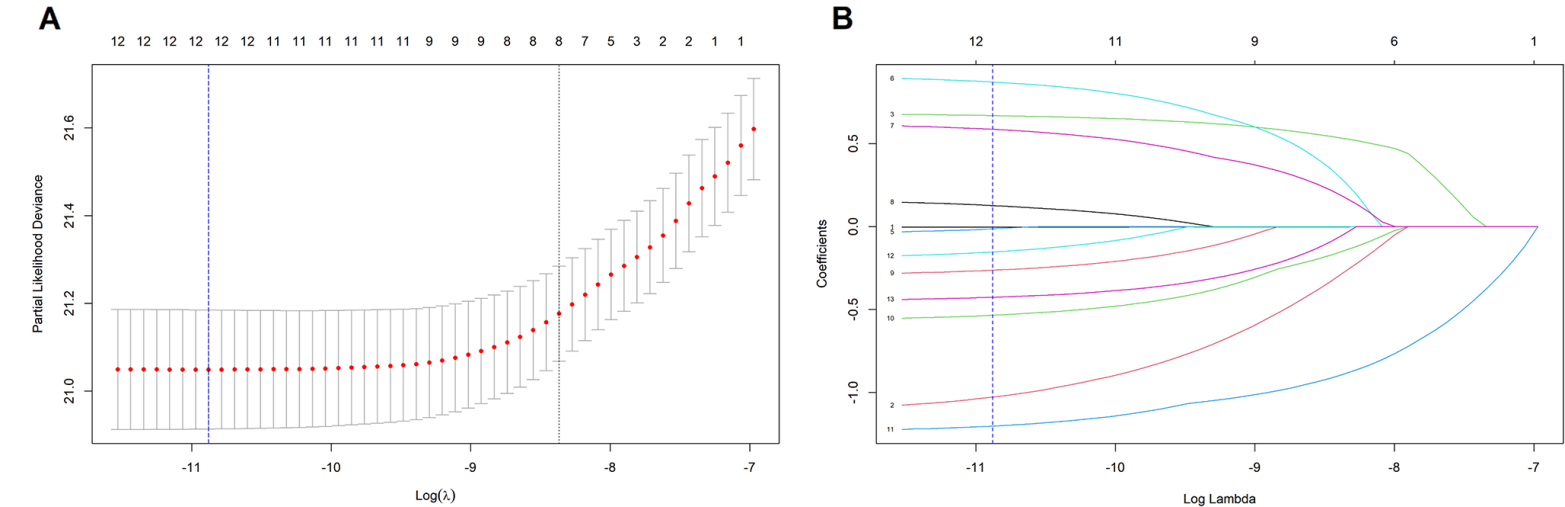
Screening of variables based on LASSO regression. (**A**) The variation characteristics of the coefficient of variables; (**B**) the selection process of the optimum value of the parameter l in the Lasso regression model by cross-validation method

**Fig. 3:**
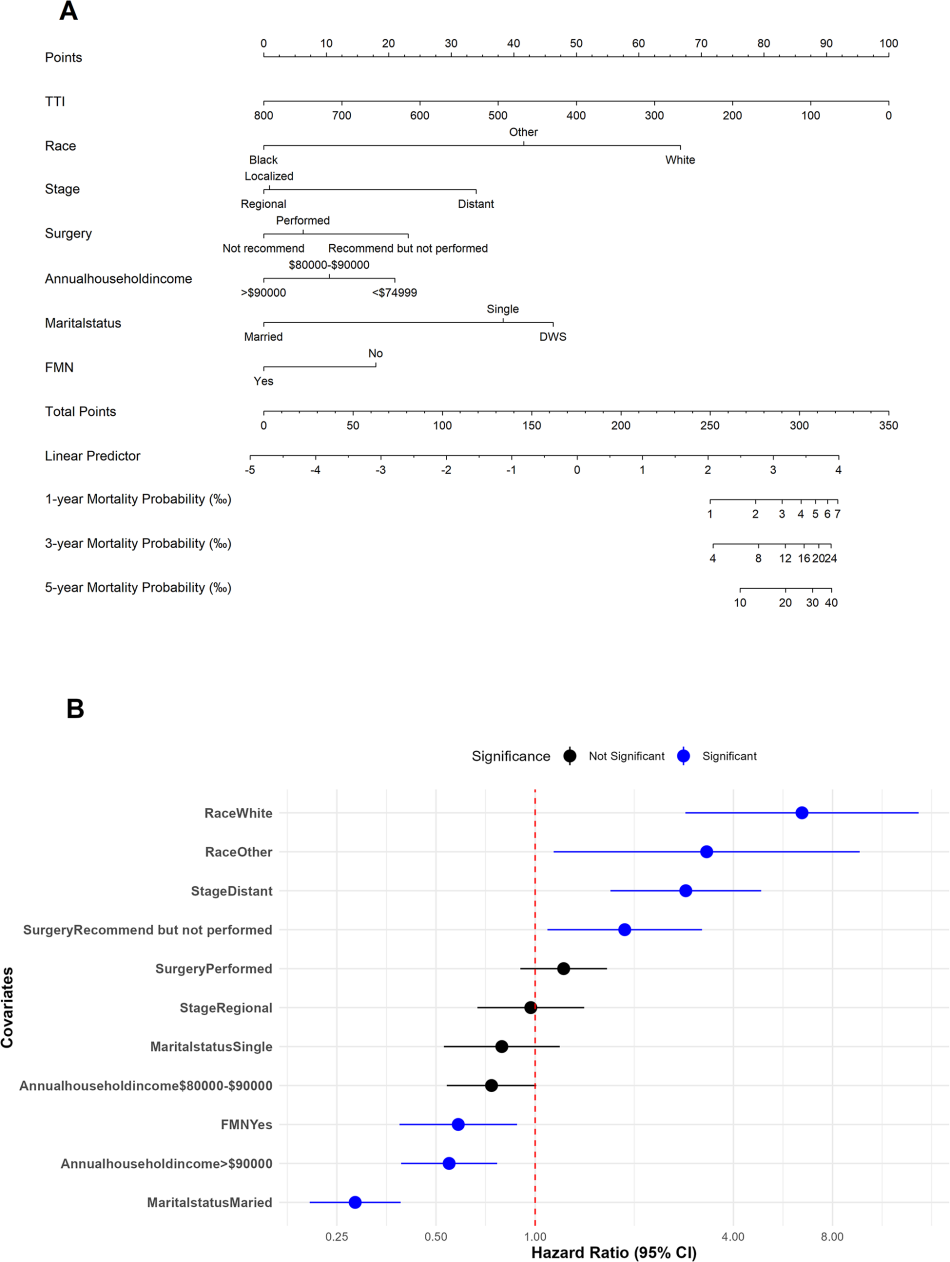
(**A**) Proposed nomogram for suicidality; (**B**) Forest plot of the model

Lambda.1se was selected as the optimal λ value for the LASSO regression, as shown in Fig. 2. Univariate and multivariate Cox proportional hazards regression models identified race, marital status, surgery, stage, annual household income, First Malignant Neoplasm (FMN), and Time of Treatment Initiation (TTI) as independent predictors of suicide among prostate cancer patients. Based on these predictors (Table 2), a nomogram and corresponding forest plot were developed to visualize and stratify high-risk patients (Fig. 3).

**Table 2:** Univariate and multivariate Cox regression models for suicide risk in the training cohort

Variables	Univariate Cox		Multivariate Cox	
	HR(95%)	*P*-value	HR(95%)	*P*-value
**Age(years),** *n* **(%)**				
≤ 64	Reference		Reference	
> 64	1.36(1.04,1.79)	0.02	1.27(0.95,1.69)	0.102
**Race**, **n(%)**				
Black	Reference		Reference	
White	5.28(2.34,11.88)	< 0.001	5.75(2.53,13.04)	< 0.001
Others	2.15(0.74,6.18)	0.157	2.80(0.96,8.21)	0.060
**Stage**, **n(%)**				
Localized	Reference		Reference	
Regional	1.04(0.72,1.48)	0.851	0.98(0.67,1.42)	0.905
Distant	3.00(1.79,5.00)	< 0.001	2.47(1.45,4.23)	< 0.001
**Surgery**, **n(%)**				
Not recommend	Reference		Reference	
Recommend but not performed	2.05(1.20,3.50)	0.009	1.88(1.09,3.22)	0.023
Performed	1.13(0.86,1.50)	0.378	1.26(0.92,1.72)	0.146
**Annual household income**, **n(%)**				
<$74,999	Reference		Reference	
$80,000-$90,000	0.71(0.52,0.97)	0.031	0.82(0.58,1.15)	0.254
>$90,000	0.52(0.37,0.72)	< 0.001	0.62(0.43,0.89)	0.010
**Marital status**, **n(%)**				
SDW	Reference		Reference	
Maried	0.30(0.22,0.42)	< 0.001	0.28(0.21,0.39)	< 0.001
Single	0.76(0.50,1.13)	0.174	0.83(0.55,1.25)	0.377
**Residence**, **n(%)**				
Non-metropolitan	Reference		Reference	
Metropolitan	0.55(0.40,0.76)	< 0.001	0.800(0.55,1.16)	0.236
**FMN**, **n(%)**				
No	Reference		Reference	
Yes	0.56(0.37,0.84)	0.005	0.64(0.42,0.96)	0.032
**Radiaotherapy**, **n(%)**				
No/Unknown	Reference		-	-
Yes	0.77(0.59,1.02)	0.064	-	-
**Chemotherapy**, **n(%)**				
No/Unknown	Reference		-	-
Yes	2.44(0.90,6.58)	0.078	-	-
**TTI(days)**	0.995(0.993,0.998)	< 0.001	0.997(0.994,0.999)	0.015

Abbreviations SDW, Separated/Divorced/Widowed; FMN, First Malignant Neoplasm; TTI, Time of Treatment Initiation

### Model Performance and Validation

**Fig. 4:**
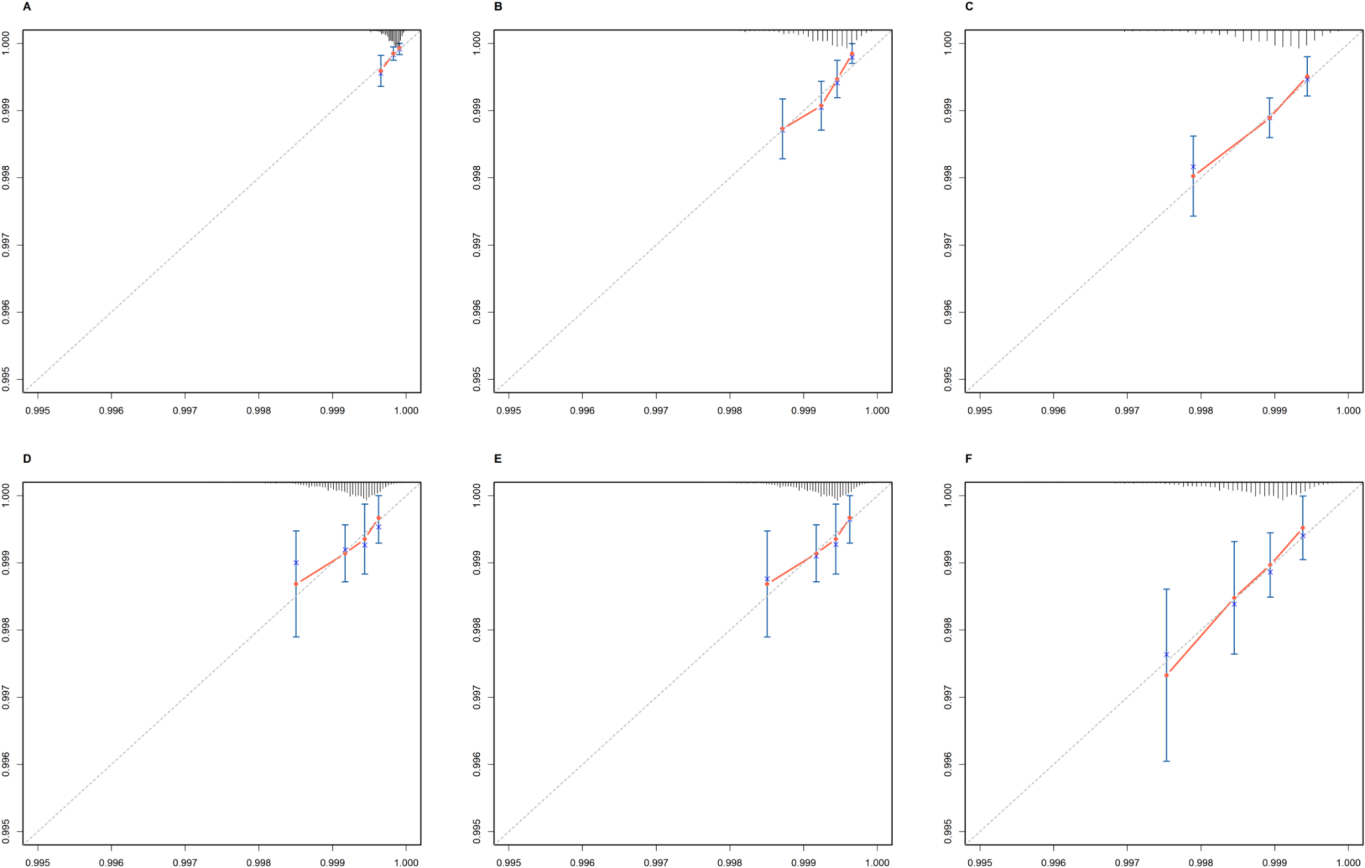
The calibration plot for prediction in the training and validation cohorts. (**A**-**C**) shows the 1-year, 3-year and 5-year endpoints in the training cohort, and (**D**-**F**) shows the 1-year, 3-year and 5-year endpoints in the validation cohort

The performance and internal validation of the nomogram were evaluated using multiple metrics. In the training cohort, the concordance index (C-index) was 0.746 (95% CI: 0.712–0.779), while in the validation cohort, the bootstrapped C-index was 0.703 (95% CI: 0.6452–0.74), indicating good predictive accuracy. Calibration curves for 1-year, 3-year, and 5-year predictions demonstrated strong concordance between predicted probabilities and actual outcomes in both cohorts, confirming the model’s reliability (Fig. 4).

### Discriminative Ability and Risk Stratification

**Fig. 5:**
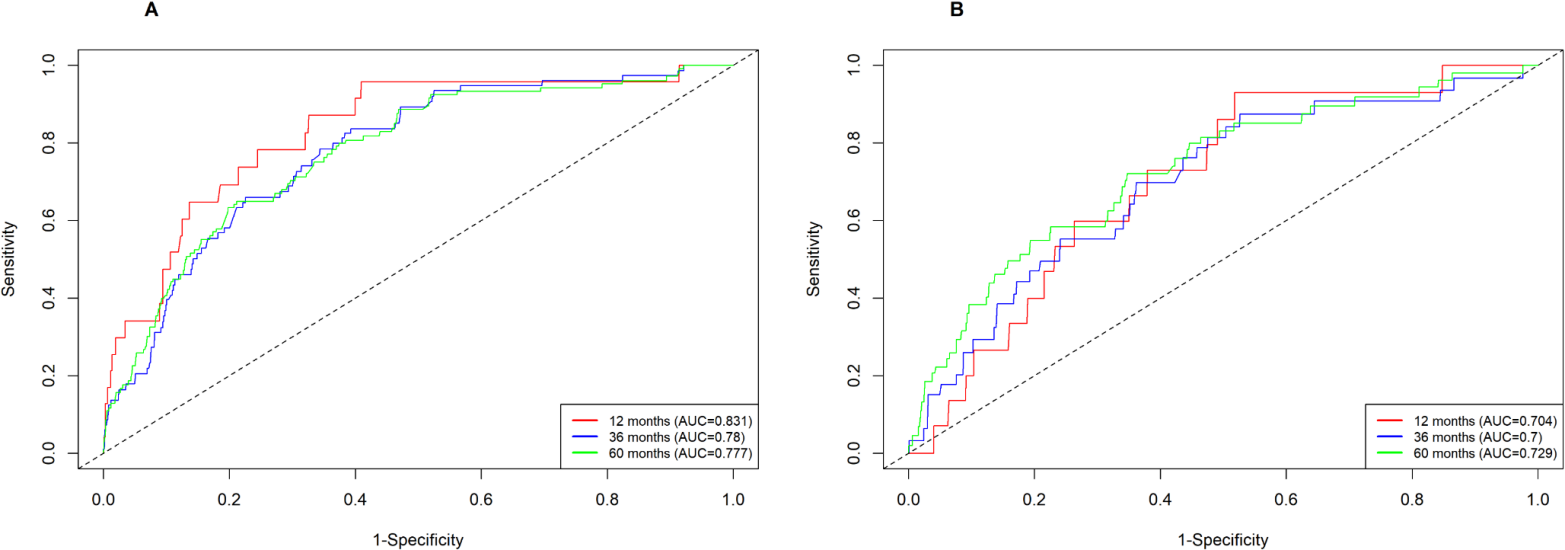
Time-dependent ROC curves for the nomogram model in the training cohort (**A**) and validation cohort (**B**)

**Fig. 6:**
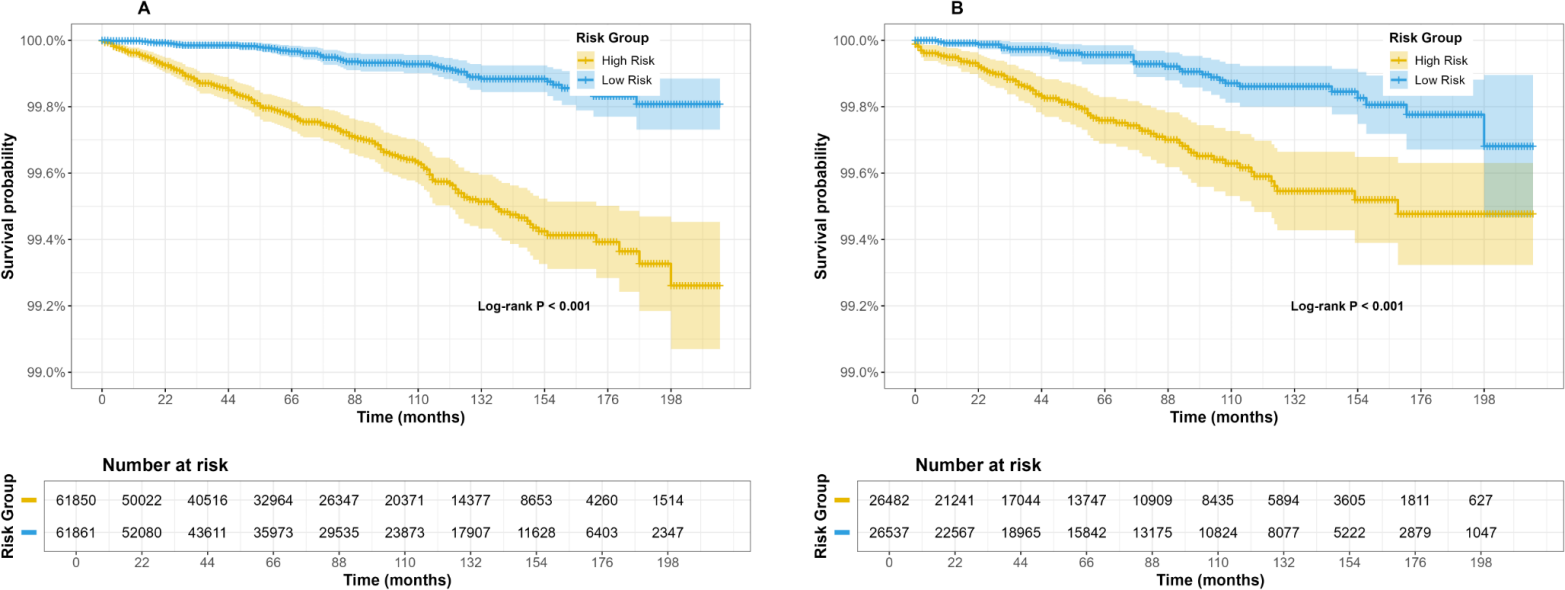
Kaplan-Meier curves of prostate cancer patients in the training cohort (**A**) and validation cohort (**B**) according to risk grouping

The model’s discriminative ability was assessed using time-dependent ROC curves, with AUC values of 0.831 (1-year), 0.780 (3-year), and 0.777 (5-year) in the training cohort (Fig. 5A), and 0.704 (1-year), 0.700 (3-year), and 0.729 (5-year) in the validation cohort (Fig. 5B). These values reflect the model’s effectiveness in distinguishing between high-risk and low-risk patients. We used an established nomogram to calculate the total points for each patient. By applying ROC curve analysis, we determined an optimal cut-off point of 306.5, and Kaplan-Meier survival analysis revealed significant differences in suicide survival rates between high- and low-risk groups in both cohorts (Fig. 6). Overall, these findings indicate that the nomogram demonstrates strong performance and stability, making it a valuable tool for identifying prostate cancer patients at high risk of suicide.

## Discussion

Suicide among prostate cancer patients often results from multiple risk factors accumulated over a lifetime, with chronic diseases, particularly malignant tumors, playing a significant role in triggering suicidal behavior. Prostate cancer imposes substantial physical and psychological burdens, exacerbating vulnerabilities and increasing suicide risk [17]. Comorbidities, severe pain, and the impact of cancer treatments further compound these risks [18]. Given the unique risk profiles of prostate cancer patients, developing tailored suicide risk assessment models for this population is crucial. This study is the first to use machine learning methods to quantify suicide risk specifically in this patient group. Our nomogram integrates seven specific risk factors: age, race, marital status, stage, surgery, annual household income, and FMN. As a more understandable and user-friendly alternative, the nomogram provides a visual representation of risk, offering clinicians a practical tool to identify high-risk patients and implement timely interventions [19, 20].

A critical aspect revealed by our study is the significant disparities in suicide risk related to race among prostate cancer patients. Zeng et al. found that black patients exhibited higher rates of death from prostate cancer, with adjusted hazard ratios (HRs) of 1.40, indicating significant racial disparities in mortality rates even after adjustments for various factors [21]. The correlation between race and suicide among prostate cancer patients is multifaceted, encompassing socioeconomic factors, access to healthcare, and cultural differences. Black men often face socioeconomic challenges that limit access to high-quality healthcare and early detection services, leading to advanced disease stages at diagnosis. This may be associated with poorer prognosis and increased psychological distress, contributing to higher suicide rates [22]. Cultural stigmas surrounding mental health and cancer within Black communities may also prevent individuals from seeking timely psychological support, exacerbating feelings of isolation and hopelessness. Additionally, experiences of systemic racism and discrimination can lead to chronic stress, negatively impacting mental health and increasing the risk of suicidal ideation among Black prostate cancer patients [23, 24].

Our findings also corroborate the elevated suicide risk among patients with distant stage cancer. Heinrich et al. conducted a meta-analysis of 62 studies, highlighting the increased suicide risk in this patient group and the need for specialized care to mitigate this risk [25, 26]. Similarly, Carlsson et al. found a significant correlation between distant cancer stages and higher suicide rates [27], suggesting a link between poor cancer prognosis and adverse mental health outcomes. Patients with distant stage cancer often experience greater physical and emotional distress, poorer prognosis, and increased treatment burdens. This stage typically required aggressive treatments with significant side effects, resulting in a reduced quality of life. The reality of limited life expectancy can lead to existential distress [28, 29]. Social isolation, loss of independence, and the financial strain of prolonged treatment further exacerbate the psychological burden [30, 31]. These factors can result in feelings of hopelessness and despair, making suicide appear as an escape from unmanageable suffering.

Patients for whom surgery was recommended but not performed are at a significantly higher risk of suicide compared to others. The multivariate Cox analysis showed a hazard ratio (HR) of 1.88 with a p-value of 0.023. Surgery, as a treatment option, can have varied psychological impacts depending on whether it is recommended, performed, or not recommended. Patients who undergo surgery may experience postoperative complications, chronic pain, and changes in body image urinary and sexual function, leading to depression and anxiety [32, 33]. Conversely, those for whom surgery is recommended but not performed can face profound psychological challenges. The heightened anxiety stemming from uncertainty about their disease progression, coupled with the perceived loss of a potential curative opportunity, can severely affect their mental well-being. This group of patients may feel particularly isolated and overlooked by the medical system when a planned surgical intervention is delayed or canceled. Such perceptions can contribute to intense feelings of abandonment and worthlessness, potentially spiraling into deep despair [34].

Another significant finding is the concerning relationship between annual household income and suicide risk among prostate cancer patients. Lower-income patients are more likely to experience poor social and family well-being, a critical factor in mental health. The stress of managing a chronic illness like cancer, compounded by financial strain, can lead to severe psychological distress. Studies have shown that men with low household income have 3.77 times higher odds of poor social and family well-being among prostate cancer survivors [35]. Financial hardship can limit access to necessary medical treatments and support services, increasing anxiety about disease progression and treatment outcomes. This socioeconomic disparity exacerbates psychological stress and contributes to a sense of hopelessness and despair, ultimately increasing the risk of suicide [36, 37]. Additionally, the stigma associated with financial instability can lead to feelings of worthlessness and social exclusion. These factors together significantly increase the risk of suicidal ideation and behavior [38].

Marital status also plays a crucial role in suicide risk among prostate cancer patients. Being married significantly reduces the risk of suicide compared to being SDW, or single, highlighting the protective effect of marriage [39]. Studies have shown that married prostate cancer patients benefit from better psychological support and social stability, leading to lower suicide rates [40]. Interestingly, single men also exhibit an increased risk of developing high-grade cancers [41]. Marriage provides emotional support, financial stability, and a sense of belonging, all of which are crucial for mental well-being [42]. In contrast, those who are single or SDW often lack the emotional and social support necessary to cope with their diagnosis. The loss of a partner or the stress of a separation can lead to severe emotional distress, while financial instability following a divorce or the death of a spouse can exacerbate anxiety and depression [43].

The initial diagnosis of a FMN in prostate cancer patients elicits profound psychological effects, often plunging individuals into significant emotional turmoil. This initial shock can precipitate severe psychological stress, anxiety, and depression. Patients grapple with the sudden, disruptive nature of the diagnosis, which not only alters their outlook on the future but also challenges their mental resilience, further compounded by uncertainties about future health, treatment effectiveness, and potential mortality [44, 45]. Additionally, changes in familial roles and personal relationships can evoke feelings of inadequacy and dependency, thereby undermining one’s sense of autonomy and self-worth. The loss of physical and social independence intensifies the psychological distress experienced by these patients. These factors collectively create a complex web of psychological and social challenges that amplify the impact of the diagnosis, extending its effects beyond the individual to families and social networks [46, 47].

Given these emotional challenges, it is important to examine the TTI. Cox analysis results indicate that a longer TTI has a slightly protective effect on suicide risk among prostate cancer patients. This suggests that patients who have a longer period before treatment initiation may experience reduced immediate psychological distress, potentially due to having more time to process their diagnosis and seek social or psychological support. Despite the counterintuitive nature of this finding, it highlights the complexity of psychological responses to cancer diagnosis and treatment. Currently, suicide risk among prostate cancer patients is known to be highest within the first year following diagnosis, with a gradual decline in subsequent years [48, 49]. This initial period is fraught with significant emotional and psychological challenges as patients come to terms with their new reality and impending treatment. Delayed treatment initiation can provide patients with additional time to psychologically adapt to their diagnosis, engage with mental health services, and gather social support, potentially mitigating acute distress. However, it is essential to balance this with the clinical need for timely cancer treatment. Sociodemographic and clinical factors significantly influence delays in treatment, affecting mental health outcomes [50]. While longer TTI may reduce initial psychological distress, ensuring that treatment delays do not negatively impact clinical outcomes is crucial.

This study found, through Kaplan-Meier curve analysis, that at the end of the follow-up period (approximately 16 years), the 16-year absolute suicide risk difference between the high-risk and low-risk groups was only 0.5% (99.3% vs. 99.8%). This result intuitively reflects the extremely low occurrence rate of suicide events (baseline incidence of 0.18%) and the mathematical challenges faced when predicting rare events. However, this result still holds potential significance in the fields of epidemiology and public health. We believe that in the prediction of rare events, the core value of the model lies not in providing deterministic predictions for individuals, but in optimizing screening efficiency through risk enrichment. At the end of the follow-up period, the suicide rate in the high-risk group was approximately 0.7%, in the low-risk group it was 0.2%, and in the general population, it was 0.5%. Screening for high-risk groups captured positive events with an efficiency 1.4 times higher than that of the general population. Given the large base of the cancer population, this model may play a role in rare events like suicide. Moreover, redefining “high-risk” patients as “vulnerable groups requiring priority attention” better aligns with the intended purpose and value of this model in suicide prediction. For example, if this model were embedded into electronic medical records and used to identify such groups in practice, maybe we could inform the patient by saying: “Your suicide risk is 3.5 times higher than others, but the absolute risk remains below 1%. We recommend offering free psychological counseling once a year. Do you agree?”

### Advantages and Limitations

This study leveraged a nationwide, high-quality cohort of 176,730 prostate cancer patients from the SEER database to investigate suicide risk factors. By utilizing a comprehensive set of demographic, clinical, and socioeconomic variables with long-term follow-up, this research provides a robust analysis of suicide risk in this population. As the first large-scale cohort study of its kind, it fills a significant gap in the literature. The study developed a nomogram using LASSO for feature selection and Cox proportional hazards regression, identifying key predictors of suicide risk. This model offers clinicians a practical tool for identifying high-risk patients and implementing timely interventions, thereby enhancing clinical utility and efficiency.

However, the study’s reliance on the SEER database limits it to the available data, which may lack detailed clinical and mental health information. Potential coding errors and reporting biases within the SEER database could affect the accuracy of suicide rates. Additionally, the retrospective design limits causal inference, underscoring the need for prospective studies to establish causality. Furthermore, the model’s internal validation requires external validation with independent datasets to confirm its generalizability and robustness across diverse populations and settings. Despite these limitations, this study provides a valuable predictive model for assessing suicide risk among prostate cancer patients and emphasizes the need for future research to enhance its predictive capability and clinical applicability.

## Conclusion

In conclusion, this study presents a useful predictive model for assessing suicide risk among prostate cancer patients, leveraging a large, high-quality dataset and employing advanced statistical methodologies. Despite its limitations, the study offers valuable insights and a practical tool for clinicians, while highlighting areas for future research to further enhance the model’s predictive capability and clinical applicability.

## Electronic Supplementary Material

Below is the link to the electronic supplementary material.

Supplementary Material 1

## Data Availability

No datasets were generated or analysed during the current study.

## Abbreviations

SEER: Surveillance, Epidemiology, and End Results
LASSO: Least Absolute Shrinkage and Selection Operator
ROC: Receiver Operating Characteristic
C-index: Concordance Index
ICD-O-3: International Classification of Disease for Oncology (third edition)
HHS: Health and Human Services
NCI: National Cancer Institute
IRB: Institutional Review Board
SDW: Separated/divorced/widowed
AUC: The area under the ROC curve
TTI: Time to treatment initiation
FMN: First malignant neoplasm
